# Suicide epidemic in Malawi: what can we do?

**DOI:** 10.11604/pamj.2021.38.69.27843

**Published:** 2021-01-20

**Authors:** Gift Treighcy Banda, Natasha Banda, Anthony Chadza, Chisomo Mthunzi

**Affiliations:** 1 Global Health, Department of Communications, Free Spirits Mental Health Awareness Group, Blantyre, Malawi,; 2 Queen Elizabeth Central Hospital, Department of Logistics, Free Spirits Mental Health Awareness Group, Blantyre, Malawi,; 3 The Catholic University of Malawi, Counselling, Free Spirits Mental Health Awareness Group, Blantyre, Malawi

**Keywords:** Suicide, mental health, public health, noncommunicable disease

## Abstract

Suicide continues to be a global health concern, affecting all continents. Although some studies have associated it with mental disorders such as severe depression, research also shows that a significant number of cases occur due to emerging life stresses. It is one of the leading causes of death among young people and is steady on the rise in Malawi. Malawi’s suicide cases disproportionately affect young males from rural areas. These cases are also higher than those of neighbouring countries. During the lockdown period to mitigate the impact of the COVID-19 pandemic between April and September 2020, Malawi saw a rise in suicide cases, most of which were due to the resulting financial hardship. There is need to tackle the suicide epidemic holistically, on all tiers of intervention. People need to be equipped with socially acceptable coping mechanisms which are easily adaptable to a low resource setting. There is a need for initiative to be taken in training individuals who can manage mental ill health without overwhelming the health system. The entire health system and health policies should acknowledge the importance of mental ill-health and its consequences. Malawi needs to prioritise mental health issues, realising that indeed, there is no health, without mental health.

## Commentary

Suicide is defined as the “death caused by self-directed injurious behaviour with intent to die as a result of behaviour” [1]. Suicide continues to be a serious global public health concern that is on a rapid rise and is one of the leading causes of death worldwide [1]. It is a leading cause of mortality particularly among adolescents and young adults; coming second to road traffic accidents [2]. It contributes to statistics in which every 40 seconds a person dies, accumulating 800,000 deaths annually [3]. Those that commit suicide are merely the tip of the iceberg, with more having unsuccessful attempts and even more afflicted by suicidal thoughts. Suicide attempt is “a non-fatal, self-directed potentially injurious behaviour”, which at times may not result in injury. Suicidal ideation is defined as “thinking about, considering or planning suicide” [1]. A link has been established between mental illnesses, namely severe depression and substance use disorders (which increase the episodes of suicide ideation), and suicide attempts [3]. However, there is a high rate of suicide committed impulsively due to emerging life issues such as relationship breakdown between family members or companions and financial crisis. While other countries have decriminalised suicide attempts, many have not. Malawi´s penal code states that “any person who attempts to kill himself shall be guilty of a misdemeanour” [4]. However, this criminalisation has proved ineffective as a deterrent from committing the act, as successful suicide attempts have been on the rise. This is leading to the current state regarding suicide; a national health outcry that is largely neglected, under researched and non-prioritised. Malawi lacks substantial prevalence data on suicide. Besides, continually high suicide rates have been reported over the past three years. In 2017, a study by Mwale and Mafuta revealed that the prevalence of suicide in Malawi was at 0.009%, (9 out of 100,000 people commit suicide in Malawi) [5], compared to the global rate of 11.1 out 100,000. The nation´s suicide rates are higher than those of the neighbouring countries such as Zambia and South Africa [5]. Within a period of nine months between the years 2018 and 2019, Lilongwe police recorded 128 suicide cases and 5 attempted suicide cases; giving a total of 133, with only five being females [6]. The Guardian newspaper has reported that in 2020 there is a “drastic rise” in suicide cases due to the COVID-19 pandemic that has caused an economic downturn in Malawi; slowdown in economic activities in the country since the beginning of the pandemic, where some companies have laid off staff due to lack of revenue and not enough funding to sustain its employees; resulting in the market reducing its performance and/or profits.

However, it would be safe to say that the pandemic has only highlighted a situation that was already a problem for Malawi- high unemployment rates, hand-to-mouth self-employment and the persistent poverty that continues to be on the rise. Perpetual poverty among Malawians threatens the very fabric of existence. It is estimated that about 50% of the Malawian population lives in poverty, with 20% living in extreme poverty. Suicide from impulsive life stresses is often connected with social and cultural factors such as debt and romantic relationship breakdown. Extreme poverty destabilises lives, crushes self-esteem and creates despair and this can lead to self-harm [7]. Despair and self-harm can evolve into suicide. In Malawi, the sociodemographic factors mostly associated with suicide are being male, between ages of 21-30, with low education and from rural areas [8]. Chasimpha *et al*. found that suicide due to financial cases was among the top 3 external causes of death in rural Malawi [8]. Other factors contributing to such high suicide rates in the country include lack of interventions such as psychosocial therapy, poor coping skills and/or cultural upbringing. Men rarely express their stresses or emotions despite there being proverbs such as “Mutu umodzi susenza denga” meaning that one person cannot carry a heavy burden alone. The Malawian culture doesn´t allow men to be vulnerable and express emotions during difficult times, unlike women; similarly, to most African cultures. Men are expected to be stoic breadwinners and protectors, showing no sign of “emotional weakness” such as crying. This is said to play a major role in suicide. Youth civil rights activists have blamed the rise in suicide rates on government´s lack of interest in improving welfare of the youth [9]. This includes lack of civic education at grassroots and local leaders on what mental health is (mental health literacy) and its impact towards an individual's livelihood and wellbeing. Even though Malawi has mental health institutions and private consultancies, there remains a gap of multi-professionals to strengthen the awareness on mental health, including coping skills. Currently, there are 5 medical doctors who have received special training in psychiatry from outside Malawi; psychologists; psychiatric nurses and clinical officers who are burdened with clinical work. There are very limited clinical social workers, who are mostly working in private mental health institutions or involved in consultancy.

Neighbouring countries have strategically acknowledged that mental health victims should not be criminalised but assisted accordingly. In South Africa, self-induced suicide attempt is legally not a crime, but assisting one to commit suicide is still a criminal offense [7]. Their 1997 mental health policy and 2002 Mental Health Care Act clearly indicates that the government should take responsibility to ensure that the citizens' mental health is taken care of. The country also has 3,460 out-patient mental health facilities [7]. In comparison to Malawi where communities largely depend on mental health nurses and clinical officers to deliver mental health services in government facilities, there remains a gap for efficient service-delivery. Several suggestions have been made on possible interventions required to reduce the suicide burden. At the individual level, approaches include increasing the number of available psychosocial therapists, who could have different tiers of training ranging from degrees to diploma level. This also includes having a holistic multi-disciplinary team working in synergy such as psychiatrists, psychologists, nurses, clinical social workers and auxiliary workers. The intensification of group and individual counselling on suicide ideation and attempts is also needed. Community level interventions must involve strategies to help change attitudes towards mental health related issues. This includes enhancing community awareness on mental health and health seeking behaviours through awareness campaigns, among others from grassroots to local leaders. Targeting children in the community is also required to ensure future populations are more equipped with emotional management and expression skills, especially amongst men. This can be ensured through introduction of “emotional awareness” topics and strategies to school curriculums.

The final tier of interventions includes policy changes and other deliberate steps to be taken by authorities. A commonly cited intervention in Malawi includes the removal of suicide from the penal code acknowledging that suicide attempts are a symptom of illness rather than a crime. Other suggestions include deliberate steps addressing existing gender norms most of which, like toxic masculinity, contribute to suicide statistics. This is through ensuring all programs and policies are intentional in addressing these norms through gender transformative approaches. Finally, prevention of suicide is also achieved through indirect methods including strategies to improve the socioeconomic status of the average citizen and reducing the unemployment levels. Improving healthcare is also vital to ensure that people can access health services, thus reducing the number of those who are physically unwell which is a contributor towards suicide. This improvement would also increase the chances of referrals by health professionals to mental health service providers. It is high time Malawi invested in training mental health workers, especially clinical psychologists who are crucial in mitigating the impact of depression and other mental health conditions. The media is another tool necessary to harness in the fight against suicide. This can be through ensuring journalists focus on their social responsibility and are deliberate particularly with reports concerning suicide. Suggestions in this area include no explicit details in reports of how suicide incidents were carried out, as well as including the different available mental health helplines at the end of such articles.

## Conclusion

The effects of the rise in suicide cases will harm the development of the nation. The current actions and health system setup are inadequate to curb the impact of the epidemic. Malawi should start prioritising mental health issues and realise that indeed, there is no health, without mental health. Intersectionality should also be considered when developing solutions.
